# A Randomized Clinical Trial Comparing the Effect of Different Haemostatic Agents for Haemostasis of the Liver after Hepatic Resection

**DOI:** 10.1155/2013/587608

**Published:** 2013-09-17

**Authors:** Farzad Kakaei, Mir Salim Seyyed Sadeghi, Behnam Sanei, Shahryar Hashemzadeh, Afshin Habibzadeh

**Affiliations:** ^1^Tabriz University of Medical Sciences, Tabriz, Iran; ^2^Imam Reza Hospital, Tabriz University of Medical Sciences, Golgasht Street, Tabriz 51666-14695, Iran; ^3^Isfahan University of Medical Sciences, Isfahan, Iran

## Abstract

*Introduction*. Operative blood loss is still a great obstacle to liver resection, and various topical hemostatic agents were introduced to reduce it. The aim of the current study is to evaluate effects of 3 different types of these agents. *Methods*. In this randomized clinical trial, 45 patients undergoing liver resection were assigned to receive TachoSil, Surgicel, and Glubran 2 for controlling bleeding. Intraoperative and postoperative findings were compared between groups. *Results*. Postoperative bleeding (0 versus 33.3%, *P* = 0.04) and drainage volume first day after surgery (281.33 ± 103.98 versus 150.00 ± 60.82 mL, *P* = 0.02) were significantly higher in Surgicel than in TachoSil group. Postoperative complications included bile leak (3 cases in Surgicel, 1 case in TachoSil and Glubran 2), noninfectious collection (2 cases in TachoSil and Surgicel and 1 case in Glubran 2), perihepatic abscess, and massive hematoma around hepatectomy site both in Surgicel group. There was no death during the study period. *Conclusion*. Due to higher complications in Surgicel group, its application as hemostatic agent after liver resection is not recommended. Better results in TachoSil in comparison to the other two are indicative of its better efficacy and superiority in controlling hemostasis.

## 1. Introduction

Advances in surgical technique have reduced the occurrence of postoperative complications following liver resection [1] and resulted in low surgical mortality and morbidity rates in high-volume centers [2–4]. Surgical techniques and devices to facilitate haemostasis have been developed in the last decades and have minimized operative risks of liver resection [1, 2]. 

During liver resection, the control of bleeding is a major concern. Despite the improvements in anatomic resection and dissection techniques, operative blood loss remains a major problem affecting the prognosis of patients undergoing liver resection [2, 5]. Nevertheless, a parenchymal transection of the liver tissue is always associated with some degree of bleeding due to the division of small blood vessels which cannot be isolated and ligated [6, 7].

In order to control diffuse bleeding and to prevent intraperitoneal complications attributed to bleeding, various topical products are used when the conventional methods, such as suture, ligation, or argon beam coagulation, fail. Currently, there are numerous products on the market which are promising a successful outcome for hemostasis. These products include gelatin, collagen, oxidized regenerated cellulose, fibrin sealant glues, and synthetic glues [7–11].

TachoSil (fibrin sealant glue) and Surgicel (cellulose based hemostat) are among those with considerable success [11]. Glubran 2 (synthetic cyanoacrylate glue) is a newly developed agent with promising results in various surgeries [12, 13]. Due to its success, it is possible to be used as a hemostatic agent in liver resection. These products have various differences considering efficacy, expenses, and mechanism of action (which will be discussed later). However, because these two agents (Tachosil and glaubran 2) are relatively new, there is no study evaluating and comparing the efficacy of these three hemostatic agents in liver resection, and there are many controversies about the ideal agent for liver haemostasis right now. We aim to compare intraoperative and postoperative findings in patients undergoing liver resection using TachoSil, Surgicel, or Glubran 2. We used these three agents because of their availability in our center and different mechanisms of action.

## 2. Methods

### 2.1. Study Population

In this randomized clinical trial, 45 patients (18–75 years old) undergoing liver resection for any underlying disease and with resectable mass in Imam Reza Hospital, Tabriz, Iran, during a six-month period from January 2012 till January 2013 were randomly assigned to receive application of cyanoacrylate glue (Glubran 2, GEM S.R.L., Viareggio, Italy) in the aerosol form or be treated by TachoSil (TachoSil, Takeda Pharmaceuticals International GmbH, Zurich, Switzerland) or Surgicel (Surgicel, Ethicon Inc., a Johnson's and Johnson's company). Operability of the patients was fully evaluated before procedure by abdominal triphasic computed tomographic (CT) scan, Duplex ultrasonography of liver vessels, chest X-rays, and chest or brain CT scans when indicated. Patients were randomly assigned to these 3 groups by a web-based calculator available in this web address: http://www.randomizer.org. Randomization was continued till the number of patients in each group reached 15. We could not increase the number of our patients because of the invasive nature of these procedures, and also it was the first time that Glubran 2 was used in liver resection procedure. 

### 2.2. Haemostatic Agents

Glubran 2 is a synthetic surgical glue, Communauté Européenne (CE) certificated for internal and external use, with haemostatic, adhesive, sealer, and bacteriostatic properties. When used in moist environment, it quickly polymerizes into a thin elastic film which has high tensile strength and firmly adheres to the anatomy of the tissue on which it is applied. Once it is polymerized, Glubran 2 acts as a bioinert material. We used 1 package of 1 mL Glubran 2 for each patient.

TachoSil, a sterile, ready-to-use, absorbable surgical patch consisting of an equine collagen sponge coated with human fibrinogen and human thrombin measuring 9.5 × 4.8 × 0.5 cm, was applied on the resection surfaces after being moistened with physiological saline. The yellow-coated side (active side) of the patch was held against the resection surface for 3 minutes to ensure uniform contact. The resection site(s) had to be covered ≥1 cm beyond its margin, and if >1 patch was needed, they had to overlap. We used only one package of TachoSil for each patient. 

Surgicel absorbable hemostat is a sterile absorbable knitted fabric prepared by the controlled oxidation of regenerated cellulose. The fabric is white with a pale yellow cast and has a faint caramel-like aroma. It is strong and can be sutured or cut without fraying. After Surgicel has been saturated with blood, it swells into a brownish or black gelatinous mass which aids in the formation of a clot, thereby serving as a haemostatic adjunct in the control of local hemorrhage. When used properly in minimal amounts, Surgicel hemostat is absorbed from sites of implantation with practically no tissue reaction. In this study, a 10 × 10 cm product was used for each patient. 

### 2.3. Resection Method and Inclusion and Exclusion Criteria

All patients with resectable liver lesions of any size during this period were included in this study. Liver resection were done by “clamp and sew” technique following the anatomic cut surfaces without the use of any specialized liver cutting system such as Waterjet systems. Patients with oozing from the resection site despite proper homeostasis effort (ligation, suture ligation, argon beam coagulation, or electro-cauterization) were included. Patients with chronic liver disease, coagulopathy not corrected with treatment before the surgery, death during surgery, operation discontinuation due to severe acidosis or coagulopathy, acute liver failure diagnosed with severe acidosis, and severe uncontrolled INR were excluded. Patients in need of resurgery due to bleeding or bile leak from liver other than resection site were also excluded. 

### 2.4. Ethical Issues

This clinical trial was approved by the Ethics Committee of Tabriz University of Medical Sciences, Tabriz, Iran, and was also registered in the Iranian Registry of Clinical Trials, and informed written consent was obtained from each patient before surgery. 

### 2.5. Objectives

The primary objective was to compare time to hemostasis between groups. The largest resection area (target wound) was assessed for time to hemostasis. Hemostasis was achieved when there was no visible bleeding from the resection wound. Counting the time to hemostasis began when TachoSil, Surgicel, or Glubran 2 was applied. Another treatment method was used or repeated if hemostasis was not achieved after 5 minutes. 

Secondary end points were evaluated with special emphasis on the total drainage volume through the Jackson-Pratt drains (which were inserted at the end of operation in the resection site), the total postoperative duration of drainage, the measurement of total volume of transfused blood products, and also by abdominal ultrasonography 2 days after operation. The operative and clamping techniques used, segments resected, and hemostatic measures were recorded. Blood loss was calculated by recording the blood substitute administered and total number of sponges used and total amount of blood in the suctions.

Age, gender, type of hepatectomy, operation time, operative blood loss, and postoperative complications (bleeding, bile leakage, and wound infection) were compared in the three groups. 

### 2.6. Blinding

Blinding for surgeons was not possible owing to the nature of the used materials' consistency (spongy TachoSil knitted fabric Surgicel and liquid Glubran 2) and their packages. The postoperative assessors were completely blinded to which agents were used for each patient.

### 2.7. Data Analysis

All data were analyzed using SPSS statistical package version 16.0 (SPSS Inc. Chicago, IL, USA). Continuous data with normal distribution are given as mean ± standard deviation, otherwise as median. Categorical variables were compared by *x* ^2^. 

The given data were compared between groups using one-way ANOVA. Student's *t*-test was used for comparisons between groups in pair. A *P* value of 0.05 or less was considered significant. 

## 3. Results

In this study, 45 patients undergoing liver resection were randomly assigned to receive TachoSil (*n* = 15), Surgicel (*n* = 15), and Glubran 2 (*n* = 15) for controlling oozing at the end of the surgery. Indications for liver resections are shown in Table 1. Hemangioma and hepatocellular carcinomas were the most common causes for liver resection. There were 3 cases of hilar cholangiocarcinoma, which underwent right hepatectomy in TachoSil group and left hepatectomy in Surgicel group. 

Table 2 demonstrates baseline findings between groups. Patients were matched for demographic findings. There was also no difference between groups in hemoglobin levels before surgery. 

Intraoperative and in-hospital findings are shown in Table 3. There was no significant difference between groups and in two-by-two evaluation in intraoperative findings (*P* = NS), but in bleeding rate after homeostasis. In two by two evaluation, the difference was significant only between TachoSil and Surgicel groups (*P* = 0.04). During postsurgery admission, there were significant differences between groups in mean blood drainage volume in the first day after surgery. Surgicel group had significantly higher first day drainage volume in comparison to TachoSil (*P* = 0.02), but the difference between Glubran 2 with Surgicel and TachoSil was not significant (*P* = NS).

Bile leakage occurred in 5 cases including one in each group which was managed with percutaneous drainage (Table 3); one minor leakage (less than 100 mL/day) in Surgicel patients was resolved with the drain implanted during surgery in three days. All leakage occurred in cases with left or right hepatectomy. There was also one major bile leakage with more than 500 mL/day in a patient with segmentectomy in Surgicel group which was managed with sphincterotomy by endoscopic retrograde cholangiopancreatography (ERCP).

FFP was transfused during postoperative period because of coagulopathy only in one patient (6.7%) in TachoSil group. The patient had undergone right hepatectomy because of metastasis from colorectal cancer. 

Resurgery due to bleeding from the liver resection site was not needed in any of the cases. Noninfectious collection was also observed in 5 patients during control ultrasonography, including one left hepatectomy in Glubran 2 group, one left hepatectomy and one segmentectomy in Surgicel, and one right hepatectomy and one segmentectomy in TachoSil group. These were managed conservatively. 

There were also two major complications in Surgicel group; perihepatic abscess (defined by frank pussy aspirate, positive bacterial culture, and patient's fever) in a patient who underwent segmentectomy was managed with percutaneous drainage. There was also a massive hematoma (750 cc) around hepatectomy site in a case with right hepatectomy that was not drained with the implanted drain and was managed with percutaneous drainage two weeks later. 

Mean hospital stay was 7.46 ± 2.79 in TachoSil, 8.13 ± 3.35 in Surgicel, and 8.80 ± 3.50 in Glubran 2 group. The difference was not significant between the groups (*P* = NS).

## 4. Discussion

TachoSil has been used in different surgeries, and its efficacy is well established [11]. Surgicel is a cellulose based hemostatic agent which is used in controlling minor bleedings such as oozing from liver or lung cut surfaces with acceptable efficacy [11]. The other product is Glubran 2, an n-butyl cyanoacrylate glue with high evidence of safety and efficacy in experimental and laboratory studies [14, 15] as well as in some surgeries [12, 13]. However, it has not been evaluated for hemostatic purposes after liver resection. This study is the first study evaluating the efficacy of these three agents in controlling bleeding after liver resection. We used time to hemostasis as the primary end point and postoperative bleeding, bile leakage, collections, and infections as the secondary end points for comparing the efficacy of these three agents. Our observations show relatively better results for TachoSil in comparison to Surgicel and Glubran 2, but the difference was not statistically significant, and the statistical power of our results was too low because of the small sample size due to our limitations. Postoperative complications were most possible to be seen in Surgicel with less incidence in TachoSil group, although the difference between groups was not significant.

Briceño et al. [3] observed a better result in case of using TachoSil; surgeries using TachoSil had less drainage volume, less transfusion, less moderate to severe complications, and lower hospital stay in comparison to groups that did not receive TachoSil. 

In a study on bovine, Takács et al. [16] observed less time to hemostasis in TachoSil in comparison to Surgicel. In our study, bleeding after hemostasis and mean first postoperative day blood drainage volume in TachoSil were significantly lower than Surgicel. However, Zacharias and Ferreira [17] found a similar complication rate and mean hospital stay in TachoSil and Surgicel groups; however, TachoSil group had a little higher hospital stay and less major complications. This study did not recognize any of them superior to the other. In our study, hospital stay in TachoSil group was lower than Surgicel. 

In the only study evaluating cyanoacrylate products and Surgicel in twelve sheep, Ellman et al. [18] observed better efficacy for cyanoacrylate products. 

There are some case reports about complications due to Surgicel use including granuloma, foreign body reaction, and neurologic complications [11], as well as abscess [19]. In this study, complications in Surgicel group were bile leak in 3 cases, noninfectious collection in 2 cases, and perihepatic abscess and massive hematoma around hepatectomy site each in one patient. In our opinion, lower biocompatibility of Surgicel compared with TachoSil will result in delayed absorption of this cellulose containing material and its future infectious and inflammatory complication. The higher rate of complications in Surgicel use questions its applicability in liver resection. 

Despite better results in TachoSil and higher complications in Surgicel group, overall, the differences between groups were not significant, and none of them has any significant superiority to others. However, it is important to consider the expenses and cost of each agent before choosing one of them as hemostatic agent. In our study, all these product were purchased from local distributors. Each products price was as follows: Surgicel (10 × 10 cm) 34 US$, TachoSil (9.5 × 4.8 × 0.5 cm) 145 US$, and Glubran 2 (1 mL) 270 US$. We used only one package of each of these agents for each patient. 

Glubran 2 is the most expensive topical agent with no significantly better efficacy, and Surgicel has higher complications unlike its lower price. Considering better results of TachoSil, it is possible to consider TachoSil as the best option for hemostasis control after liver resection; however, further studies are needed to confirm these findings because of our small group and diversity of our patients (segmental versus major hepatectomies, malignant versus benign conditions, and long versus short time operations). We use hemostatic agents only when oozing after mechanical hemostasis continues. Many times, we do not need any of these agents for hemostasis if we follow strict anatomical plains for liver resection. We have performed 49 liver resections in the same period without the need of any hemostatic agents. In our experience, none of these hemostatic agents could be used for stopping major bleeding from liver vasculature especially in trauma patients. Due to our inclusion criteria (continuous oozing after surgical hemostasis), we did not include these patients in our study.

## 5. Conclusion

The results of the current study are indicative of slight differences in hemostasis control of TachoSil, Surgicel, and Glubran 2. Due to higher complications in Surgicel group, although, less expensive than the other two agents, its application as a hemostatic agent after liver resection is not recommended. Better results in TachoSil in comparison to the other two are indicative of its better efficacy and superiority in controlling hemostasis.

## Figures and Tables

**Table 1:** Indications for liver resections.

	TachoSil (*n* = 15)	Surgicel (*n* = 15)	Glubran 2 (*n* = 15)
Hepatocellular carcinoma	3	1	2
Hilar cholangiocarcinoma	1	2	0
Adrenal cancer metastasis	1	0	0
Breast cancer metastasis	1	0	0
Colorectal metastasis	1	1	2
Biliary carcinoma	1	1	2
Hemangioma	3	3	3
Hepatic adenoma	2	2	2
Focal nodular hyperplasia	2	2	1
Unilocular hydatid cyst	2	1	0
Multilocular hydatid cyst	0	1	2

**Table 2:** Baseline findings between groups.

	TachoSil (*n* = 15)	Surgicel (*n* = 15)	Glubran 2 (*n* = 15)	*P* value
Age (years)	45.73 ± 12.20	46.60 ± 12.93	50.86 ± 10.47	NS
Gender				
Male	5 (33.3%)	6 (40%)	7 (46.7%)	NS
Female	10 (66.7%)	9 (60%)	8 (53.3%)	
Hemoglobin (mg/dL)	12.40 ± 0.68	12.45 ± 1.40	13.11 ± 1.67	NS

NS: not significant.

**Table 3:** Intraoperative and in-hospital findings between groups.

	TachoSil	Surgicel	Glubran 2	*P* value
Intraoperative bleeding (mL)	766.66 ± 416.90	573.33 ± 281.49	653.33 ± 448.19	0.4
FFP during surgery	0	0.53 ± 0.36	0.80 ± 0.42	0.21
Total transfused packed cell during surgery (units)	4.33 ± 1.07	1.86 ± 0.92	2.13 ± 0.55	0.1
Time to homeostasis	3.00 ± 0.84	3.26 ± 1.48	2.66 ± 1.15	0.43
Suction volume (mL) after hemostasis	89.33 ± 57.37	116.66 ± 52.32	122.66 ± 57.25	0.22
4 × 4 Gauze pads used after hemostasis	3.06 ± 0.70	3.40 ± 1.12	3.25 ± 0.75	0.59
Abdominal pads used after hemostasis	0.80 ± 0.22	1.13 ± 0.23	1.16 ± 0.29	0.51
Bleeding after homeostasis	0	5 (33.3%)	2 (13.3%)	0.004*
First day drainage	150.00 ± 60.82	281.33 ± 103.98	234.66 ± 187.95	0.02*
Second day drainage	66.66 ± 25.19	92.00 ± 28.58	75.33 ± 22.76	0.77
Third day drainage	10.66 ± 4.62	5.33 ± 2.36	18.00 ± 7.63	0.25
Time to extract the drain (day)	3.66 ± 0.97	4.06 ± 0.70	5.26 ± 2.89	0.06
Bile leak	1 (6.7%)	3 (20%)	1 (6.7%)	0.4
Packed cell during hospitalization (units)	5 (33.3%)	4 (26.7%)	2 (13.30%)	0.6

*P is two sided significant. FFP: fresh frozen plasma.
